# Preparation of *N*-MG-modified PVDF-CTFE substrate composite nanofiltration membrane and its selective separation of salt and dye†

**DOI:** 10.1039/d4ra00359d

**Published:** 2024-04-18

**Authors:** Xinyu Pan, Jian Pan, Zhuoqun Li, Wenqiang Gai, Guangshun Dong, Min Huang, Lilan Huang

**Affiliations:** a School of Materials Science and Engineering, Shandong University of Technology No. 266 West Xincun Road, Zhangdian District Zibo 255000 China panjian@sdut.edu.cn huanglilan_sdut@163.com

## Abstract

Poly(vinylidene fluoride-*co*-chlorotrifluoroethylene) (PVDF-CTFE) is considered an ideal membrane material for the treatment of complex environmental water due to its exceptional thermal stability and chemical resistance. Thus, to expand its application in the field of nanofiltration (NF) membranes, in this study, *N*-methylglucamine (*N*-MG) was used to hydrophilically modify PVDF-CTFE, overcoming the inherent hydrophobicity of PVDF-CTFE as a porous substrate membrane, which leads to difficulties in controlling the interfacial polymerization (IP) reaction and instability of the separation layer structure. The –OH present in *N*-MG could replace the C–Cl bond in the CTFE chain segment, thus enabling the hydrophilic graft modification of PVDF-CTFE. The influence of the addition of *N*-MG on the surface and pore structure, wettability, permeability, ultrafiltration separation, and mechanical properties of the PVDF-CTFE substrate membrane was studied. According to the comparison of the comprehensive capabilities of the prepared porous membranes, the M4 membrane with the addition of 1.5 wt% *N*-MG exhibited the best hydrophilicity and permeability, indicating that it is a desirable modified membrane for use as an NF substrate membrane. The experiments showed that the rejection of Na_2_SO_4_ by the NF membrane was 96.5% and greater than 94.0% for various dyes. In the test using dye/salt mixed solution, this membrane exhibited a good separation selectivity (CR/NaCl = 177.8) and long-term operational stability.

## Abstract 2

*N*-Methylglucamine (*N*-MG) was introduced to poly(vinylidene fluoride-*co*-chlorotrifluoroethylene) (PVDF-CTFE) porous substrate membrane to enhance its hydrophilicity and permeability, leading to the preparation of a composite NF membrane with selective separation performance for many dyes and salts.

## Graphical abstract

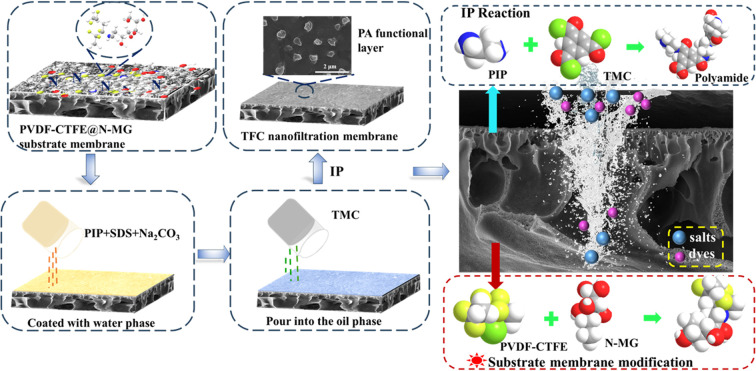

## 1. Introduction

Presently, with the growing economy, water pollution has become increasingly severe. Much substandard industrial wastewater flows into rivers and lakes, causing humans to drink contaminated water.^1,2^ Membrane separation is a separation technology that uses the permeability difference among components in a mixed system to achieve purification, screening, concentration, and other purposes. Compared with other methods, the benefits of membrane separation technology include its low energy requirements, high separation selectivity, high efficiency and low cost, and thus it has been extensively utilized in industry including wastewater treatment, saltwater desalination and drug purification.^3,4^ The membrane plays the main role in membrane separation processes, including microfiltration, ultrafiltration, nanofiltration (NF) and reverse osmosis. Among them, the molecular weight cut-off (MWCO) of NF membranes is in the range of 200 to 1000 Da, with an average pore size of around 1 nm.^5^ Surface coating or grafting, layer-by-layer self-assembly and interfacial polymerization (IP) are the primary techniques employed for the fabrication of NF membranes. The most common IP method involves the contact of two immiscible solvents on the membrane surface, where the monomers inside them react to form a functional layer. Typically, the solvents used are water solutions containing amino monomers and organic solutions containing acyl chloride monomers. IP has the advantage of fast reaction rate, by changing the monomer concentration and ratio, polymerization temperature and time and the preparation of thin-film layers with specific functions.^6,7^

The formation of the separation layer in NF membranes using the interface polymerization method is significantly affected by the porous substrate membrane. The substrate membrane pore structure, including the distribution of pore sizes, connectivity of pore channels, and overall porosity, directly affects the permeability and selectivity of the separation layer. The surface characteristics of the substrate membrane such as its hydrophilicity or hydrophobicity will affect the compatibility and adhesion of the separation layer with the substrate membrane, thereby affecting the adhesion quality and stability of the separation layer.^8^ Currently, most commercial NF membranes are made of polyether sulfone (PES), polysulfone (PSF), polyacrylonitrile (PAN), and polyvinylidene fluoride (PVDF) as the substrate membrane. PES membranes exhibit excellent oxidation resistance, anti-hydrolysis, and good mechanical properties, but they are highly hydrophobic.^9^ Furthermore, PSF membrane materials have high thermal stability and acid and alkali resistance; however, their separation accuracy is low and their anti-pollution ability is poor.^10^ Alternatively, PAN membranes have excellent hydrophilicity and anti-pollution performance, but their oxidation resistance and tensile strength are relatively poor.^11^ PVDF membranes have high cost-effectiveness, good stretching, and can be designed and installed in various shapes, but are highly hydrophobic.^12^ When used as an NF substrate membrane, the water phase monomer is difficult to penetrate, which can cause a low degree of cross-linking between the monomers in the water phase and oil phase, which is not conducive to the interface reaction and formation of the separation layer. The hydrophilic modification of the PVDF membrane surface can introduce hydrophilic groups on the porous membrane surface through specific chemical treatment or coating technology, enhancing its hydrophilicity. Chen *et al.*^13^ used polyethylene glycol dimethacrylate (PEGDMA) to graft-modify PVDF and found that the PVDF-*g*-PEGDMA membrane exhibited a decrease of about 80% in bovine serum albumin (BSA) adsorption compared with the original PVDF membrane, and the water contact angle decreased to 0°, greatly improving the hydrophilicity of the membrane. Xu *et al.*^14^ prepared a super-hydrophilic tea polyphenol/silica composite coating, which not only achieved super-hydrophilic modification on the porous PVDF membrane surface but also its inner surface, generating a modified membrane with excellent water permeability and ultrahigh water flux.

Poly(vinylidene fluoride-*co*-chlorotrifluoroethylene) (PVDF-CTFE) is a copolymer composed of vinylidene fluoride (VDF) and chlorotrifluoroethylene (CTFE).^15^ This copolymer introduces CTFE segments into the PVDF chain, exhibiting superior chemical resistance, better resistance to polar organic solvents, and higher mechanical strength compared to PVDF.^16,17^ Thus, it is an ideal material for water treatment in complex environments such as dyeing and printing wastewater. However, its hydrophobicity increases further with an increase in the relative content of C–F bonds,^15,18^ posing challenges when used as an NF substrate membrane. Traditional surface modification techniques only affect the membrane surface and do not permeate inside the membrane. Furthermore, surface modification effects are unstable, and under long-term use or complex conditions, the modified layer may experience wear, delamination, or degradation, resulting in a reduction or loss of its hydrophilic properties. In this case, blend modification offers advantages such as achieving overall modification, long-lasting effects, and resistance to external factors. Lv *et al.*^19^ incorporated hydrophilic cellulose nanocrystals (CNC) into hydrophobic PVDF membranes. They studied the effects of CNC on the morphology, hydrophilicity, permeability, and fouling resistance of the membranes. The findings indicated that the addition of CNC improved the permeability of the PVDF membranes by enhancing their hydrophilicity and optimizing their structure. Li *et al.*^20^ utilized the phase separation method to prepare PVDF/PVA hollow fiber membranes. With an increase in PVA content, the permeability of the membranes increased, while the rejection rate decreased. The hydrophilicity and fouling resistance of the PVDF membranes were improved by PVA. However, the tensile strength of the blended membranes was found to be lower than that of the original PVDF membrane. Shi *et al.*^21^ used the thermally induced phase separation method to fabricate PVDF/TiO_2_ hybrid membranes with varying quantities of TiO_2_ nanoparticles. They found that at higher concentrations, the TiO_2_ nanoparticles formed a continuous phase dispersed within the PVDF matrix. The permeability, hydrophilicity and mechanical properties of the membranes showed a tendency to initially increase, and then decrease with higher concentrations of TiO_2_ nanoparticles. The presence of C–Cl active sites in the PVDF-CTFE substrate membrane material provided feasibility for *in situ* substitution-graft modification. Hydrophilic compounds may be included in the casting solution as modifiers. A PVDF-CTFE membrane could be durably hydrophilized in a single-step intrinsic modification process through a substitution-graft reaction.^22^ This effectively solved the problems of the aggregation of the additives and decreased membrane strength and separation performance in traditional blend modification methods.

In this study, *N*-methylglucamine (*N*-MG), a hydrophilic compound with multiple hydroxyl groups, was used to react with the C–Cl active sites on PVDF-CTFE under mild reaction conditions. This reaction allowed hydroxyl groups to be immobilized on the PVDF-CTFE molecular chain, realizing a durable hydrophilic effect on the PVDF-CTFE porous membrane. The impacts of the addition of *N*-MG on the membrane structure and properties were evaluated through scanning electron microscopy (SEM), energy dispersive spectroscopy (EDS), differential scanning calorimetry (DSC), X-ray photoelectron spectroscopy (XPS), Fourier transform infrared spectroscopy (FTIR) and other characterization techniques, as well as by evaluating the hydrophilicity, permeability, pore size distribution, tensile strength and BSA separation performance of the membrane. To create a separation layer on the PVDF-CTFE porous substrate membrane surface, piperazine (PIP) and trimesoyl chloride (TMC) were used for interface polymerization. The prepared NF membrane was characterized using SEM, total organic carbon (TOC), and zeta potential testing. The NF performance and selective separation of simulated dyeing and printing wastewater were evaluated.

## 2. Experimental

### 2.1 Materials

PVDF-CTFE copolymer particles (Solef® 31 508, *M*_w_ = 270–290 kDa) were obtained from Solvay (Shanghai) Co., Ltd. 1-Methyl-2-pyrrolidinone (NMP) was bought from Tianjin Zhiyuan Chemical Reagent Co., Ltd. *N*-hexane and Na_2_CO_3_ were procured from Sinopharm Chemical Reagent Co., Ltd. BSA (68 kDa) was bought from Beijing Aoboxing Biotech Co., Ltd. *N*-MG used as an additive, polyvinyl pyrrolidone (PVP, 30 kDa) used as a pore-forming agent, sodium dodecyl sulfate (SDS) employed as a surfactant, the polymeric monomers PIP and TMC, polyethylene glycol (PEG) with various molecular weights ranging from 200 to 1000 Da for MWCO testing, salts such as NaCl, MgCl_2_, Na_2_SO_4_, and MgSO_4_, dyes such as Congo red (CR), acid fuchsin (AF), orange G (OG), methyl blue (MB), and methyl orange (MO) were all purchased from Shanghai Aladdin Biochemical Technology Co., Ltd. Deionized water was lab-made. All chemicals were used as received without further purification during the experiments.

### 2.2 Fabrication of porous substrate membranes

**Fig. 1:**
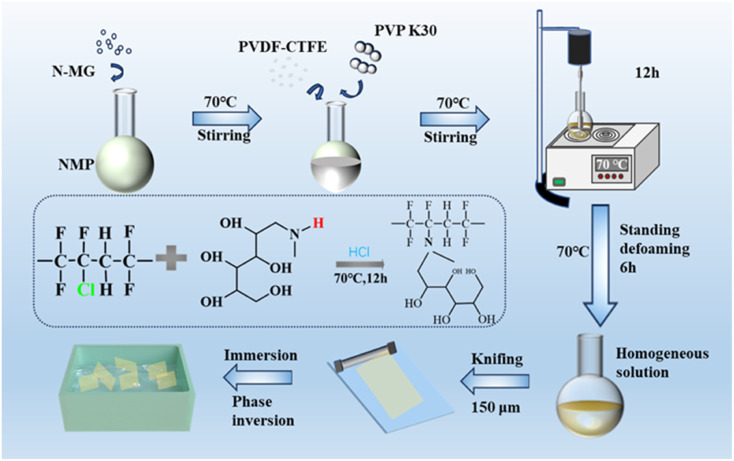
Experimental process for the preparation of PVDF-CTFE modified porous membrane by *N*-MG blending and the reaction mechanism diagram.

A certain amount of *N*-MG was placed in a round-bottom flask and an appropriate amount of NMP solvent was added. The mixture was heated and stirred in a water bath at 70 °C until completely dissolved. Then, a suitable amount of PVDF-CTFE and PVP was added to the flask. Heating and stirring were continued for 12 h until they were completely dissolved, forming a homogeneous casting solution, and then left undisturbed for 6 h to eliminate foaming. A 150 μm membrane applicator was used to evenly coat the casting solution onto a smooth and clean glass plate, and then slowly place it in deionized water for phase transformation. At last, the formed membrane was soaked in deionized water for storage, thoroughly removing excess solvents and pore-forming agents. The experimental process for the preparation of the PVDF-CTFE modified porous membranes by *N*-MG blending is shown in Fig. 1. In this experiment, the amount of *N*-MG used as a hydrophilic additive varied in the range of 0–2 wt%. The resulting porous substrate membranes were designated as M1, M2, M3, M4 and M5, as shown in Table 1.

**Table 1:** Formulation of modified porous membrane prepared by the addition of different amounts of *N*-MG

Membrane code	PVDF-CTFE content (wt%)	NMP content (wt%)	PVP content (wt%)	*N*-MG addition (wt%)
M1	16	83	1	0
M2	16	83	1	0.5
M3	16	83	1	1
M4	16	83	1	1.5
M5	16	83	1	2

### 2.3 Fabrication of composite NF membranes

**Fig. 2:**
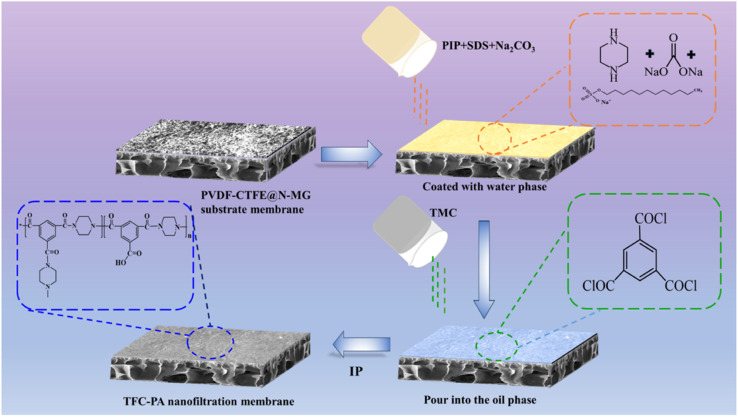
Schematic representation of the steps involved in preparing the porous PVDF-CTFE@*N*-MG modified TFC NF membrane.

To prepare the aqueous solution, 1 wt% PIP was dissolved in deionized water with 0.05 wt% SDS and 1 wt% Na_2_CO_3_. To prepare the oil phase solution, 0.25 wt% TMC was dissolved in *n*-hexane. Then, the prepared PVDF-CTFE@*N*-MG modified membrane was placed on a customized IP plate. The aqueous solution was poured onto the surface of the membrane, left to rest for 10 min, and then poured off. A rubber roller and filter paper were used to eliminate excess aqueous solution. Then, the IP reaction was initiated and the polyamide (PA) functional layer was formed by pouring the oil phase solution onto the membrane surface for 30 s. The formed thin-film composite (TFC) membrane was heated and cured in an oven at 70 °C for 10 min to complete the polymerization and remove excess solvent. After removal, it was slowly placed in deionized water for storage. The procedure for the preparation of TFC NF membrane based on the PVDF-CTFE@*N*-MG modified porous membrane is schematically presented in Fig. 2.

### 2.4 Membrane characterization

In the morphology analysis, field emission SEM (FE-SEM) (Quanta 250, FEI Co., USA) was used to evaluate the surface and cross-sectional morphology of the membranes. The elemental composition and distribution of C, F, Cl, O and N on the membrane surface were analyzed using EDS within an area of 150 × 120 μm. In the pore structure analysis, the gravimetric method was used to measure the overall porosity of the membranes. The pore size distribution and average pore size of the fabricated membranes were tested using a pore size analyzer (BSD-PB, Beishide Instrument Technology (Beijing) Co. Ltd, China). The effect of the addition of *N*-MG on the physicochemical structure of the membranes was assessed by comparing their infrared spectra by FT-IR (Nicolet 5700, Thermo Fisher Co., USA). XPS (K-Alpha, Thermo Fisher Co., USA) analysis was performed to characterize the surface chemical elements of the membrane. The thermal properties of each membrane were examined using DSC (Q100, TA Co., USA). The detailed analytical test operations and the calculations of the polymer matrix β-phase content (*F*(β)) and crystallinity (*χ*_c_) are provided in the ESI.†

### 2.5 Membrane performance test

A contact angle measuring instrument (JY-82A, Ding Sheng Co., China) was utilized to measure the static contact angle of the prepared porous substrate membranes. An electronic single fiber strength machine (YG001 N+, Hong Da Co., China) was applied to determine the elongation at break and breaking strength of the substrate membranes. The surface zeta potential of the functional layer was tested using a solid-state zeta potential analyser (Exceed 3, Anton Paar, Austria). The MWCO of the functional layer was determined using 1000 ppm PEG solutions with various molecular weights (200, 400, 600, 800, and 1000 Da) to pass through the membrane. The concentrations of PEG in both the permeate and feed solutions were analyzed using a total organic carbon analyzer (TOC-L, Shimadzu, Japan). The pore size distribution of the functional layer was obtained through the probability density function, as provided in the ESI.†

In the pure water flux, ultrafiltration and anti-fouling performance testing of the porous substrate membranes, deionized water and a 1 g L^−1^ solution of BSA were used as the test solutions successively. The membranes were pre-pressurized at 0.15 MPa for 30 min, and once the penetrating fluid reached a stable flow rate, the experiment was conducted under a pressure of 0.1 MPa. In the anti-fouling ability test, the pre-pressed membrane underwent continuous dual-cycle testing with BSA (30 min) and deionized water (60 min). The volume of the permeating fluid and the corresponding time were recorded. The absorbance of BSA in the feed and permeate solutions was measured using an ultraviolet-visible (UV) spectrophotometer (UV-1100, Mapada, China). The flux, rejection rate, flow decline ratio (FDR) and flow recovery ratio (FRR) data were calculated using the formulas in the ESI.† In the acid–alkali resistance testing of the porous substrate membranes, the test solutions were a solution of HCl (pH = 3) and NaOH (pH = 11), respectively. The membranes were soaked in the acid and alkaline solutions for 10 days, and then taken out and rinsed clean to measure the changes in their tensile strength.

In the separation performance testing of the prepared TFC NF membranes, the membrane was first pre-pressed with deionized water at 0.4 MPa for 30 min. Then, filtration separation tests were conducted at a pressure of 0.2 MPa. The filtration tests were conducted using solutions of salts (2000 ppm Na_2_SO_4_, MgSO_4_, MgCl_2_ and NaCl) and dyes (50 ppm CR, AF, OG, MO, and MB). The concentration of salts and dyes in the filtrate and feed solution was measured using a conductivity meter (DDS-307, Shanghai Yueping, China) and UV spectrophotometer, respectively. All the permeate flux and rejection rates of BSA, salts, and dyes were calculated using the formulas in the ESI.† In the stability and selective separation performance analysis, a mixture of dye (50 ppm CR or OG) and salt (2000 ppm NaCl) was used to simulate printing and dyeing wastewater. The selectivity (*α*) of dyes to salt was calculated using the formula in the ESI.†

## 3. Results and discussion

### 3.1 Morphology and structure of modified substrate membrane

**Fig. 3:**
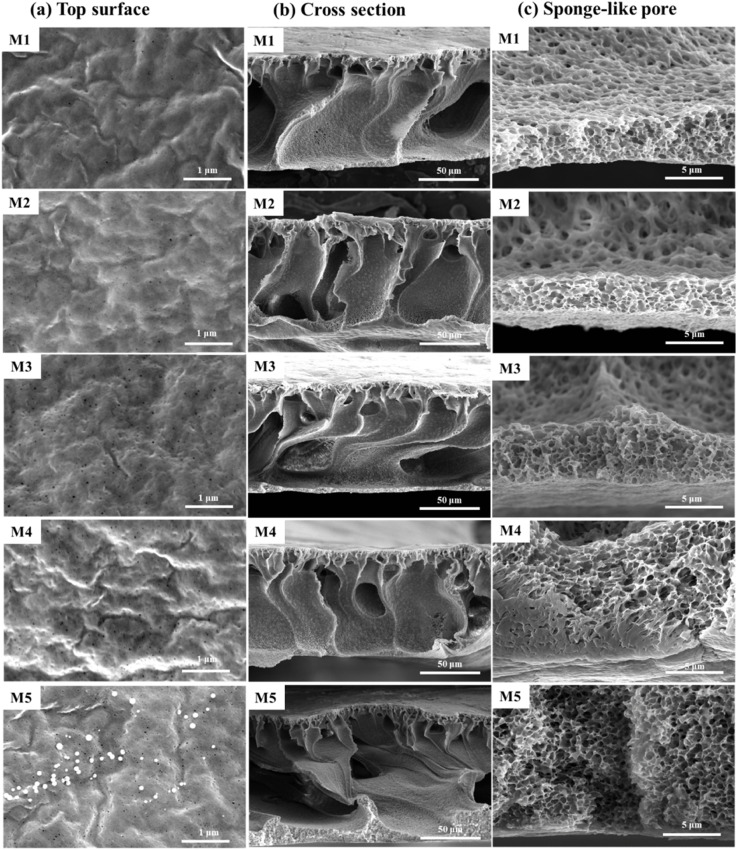
Influence of the addition of *N*-MG on the surface and cross-sectional morphology of modified PVDF-CTFE porous substrate membranes: (a) top surface, (b) cross section, and (c) sponge-like pore.

Fig. 3 presents the morphology of the PVDF-CTFE porous substrate membranes modified by the addition of different contents of *N*-MG. According to the membrane surface morphology, as shown in Fig. 3(a), the introduction of *N*-MG increased the number of micro-pores on the membrane surface. This number gradually increase with an increase in the concentration of *N*-MG. When the content of *N*-MG reached 2 wt%, white microspheres appeared on the membrane surface. The analysis suggested that the maximum *N*-MG addition was reached, and some of the *N*-MG remained undissolved (partially visible as the undissolved material on the inner wall of the flask, as shown in ESI Fig. S1†), which blocked the membrane pores. According to SEM, the surface of the prepared substrate membrane exhibited cortical and microporous structures. This was due to the phase transition occurring during the membrane formation process. From a thermodynamic perspective, the addition of *N*-MG containing –OH lowered the thermodynamic stability of the casting solution, which made it easier for the non-solvent to enter the solution and form microporous structures on the membrane surface. From a kinetic perspective, the addition of *N*-MG slightly increased the viscosity of the cast film solution, leading to a low reverse turnover rate, which is favorable for the formation of more skins on the top surface of the membrane. Additionally, the number of micropores on the substrate membrane surface increased initially, and then slightly decreased as the *N*-MG content increased, suggesting that the kinetics was now the dominant factor controlling the surface of the membrane rather than thermodynamics, as shown in Fig. 3(a). Based on the cross-sectional morphology of the membrane, as shown in Fig. 3(b), the membrane has a typical asymmetric structure with sponge-like pores at its bottom, pear-like micropores in its middle, and finger-like pores in its sublayer. This structure was primarily the result of the rapid solvent and non-solvent exchange. Liquid–liquid (L–L) demixing was facilitated by the strong polarity of NMP and the excellent hydrophilicity of PVP.^23^ The rapidly diffused non-solvent pushed the rich polymer phase to the bottom layer and pore wall, formed a sponge-like structure, and transformed the poor polymer into pear-like micropores. Additionally, as the amount of *N*-MG increased, the bottom of the spongy-shaped membrane thickened significantly. This was because the addition of *N*-MG increased the viscosity of the cast film solution, which prevented the non-solvent (water) from entering during the phase change process. This was caused by the increased viscosity of the casting liquid and the action of hydrophilic *N*-MG compounds, as seen in Fig. 3(b) and (c).

**Fig. 4:**
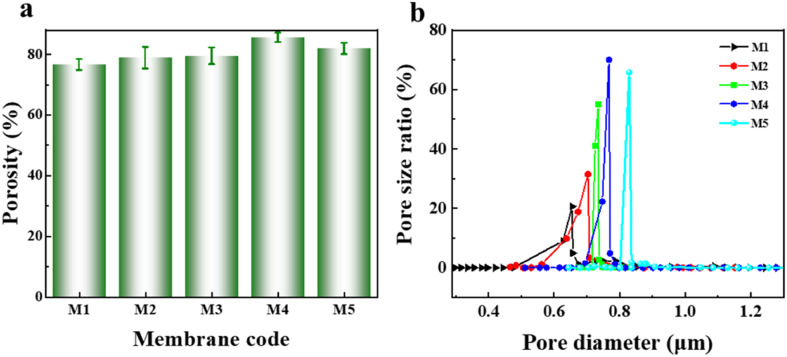
Influence of the addition of *N*-MG on the pore structure of modified PVDF-CTFE porous substrate membranes: (a) porosity and (b) pore size distribution.

Fig. 4 shows the pore structure of the PVDF-CTFE porous substrate membranes modified by varying amounts of *N*-MG. According to the overall porosity data in Fig. 4(a) and the pore size distribution in Fig. 4(b), the change in the porosity of the membranes was always higher than 75%. With an increase in the addition of *N*-MG, both the porosity and average pore size gradually increased, and the pore size distribution became narrower. When the amount of *N*-MG added reached 2 wt%, some *N*-MG remained undissolved due to reaching the maximum grafting capacity, which resulted in pore clogging, leading to a decrease in porosity and reduction in average pore size.

### 3.2 Chemical composition of modified substrate membrane surface

**Fig. 5:**
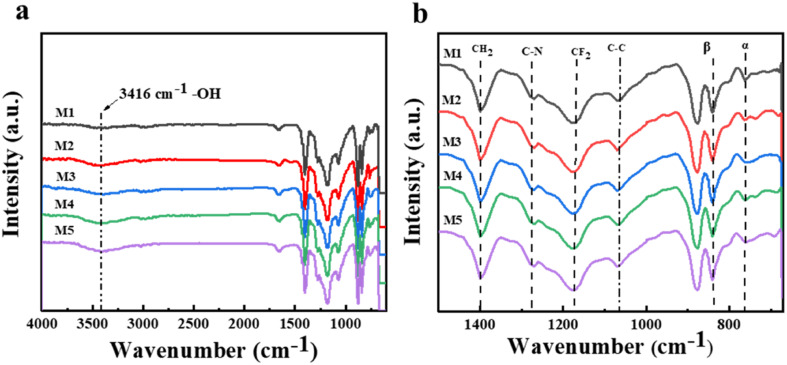
Influence of the addition of *N*-MG on the FTIR spectrum of the modified PVDF-CTFE porous substrate membranes: (a) full spectrum and (b) fingerprint region spectrum.

With the aim of further analyzing the grafting modification effect of *N*-MG on the surface of the PVDF-CTFE membranes, various techniques such as FTIR, XPS, and EDS were employed to test the chemical composition of the membrane surface. Fig. 5 illustrates the infrared spectra of the PVDF-CTFE porous membranes modified with varying amounts of *N*-MG. According to the full spectrum in Fig. 5(a), the M1 membrane without the addition of *N*-MG did not exhibit a stretching vibration peak at 3416 cm^−1^, which corresponds to –OH groups. However, after the addition of *N*-MG, characteristic peaks appeared and their intensity increased with an increase in the content of *N*-MG. In the fingerprint region displayed in Fig. 5(b), it was apparent that the characteristic peak of the C–N bond (at 1275 cm^−1^) became more pronounced with the addition of *N*-MG. This peak originated from the reaction between the active C–Cl sites in the PVDF-CTFE molecules and the –NH groups in the *N*-MG molecules.

Besides the characteristic peaks of the different functional groups in the PVDF-CTFE molecules, a transition between different phases of the PVDF-CTFE matrix could also be observed. The addition of *N*-MG gradually weakened the characteristic peak of the α phase (at 762 cm^−1^) and relatively enhanced the characteristic peak of the β phase (at 840 cm^−1^), indicating a transition process from the α phase to the β phase in the PVDF-CTFE matrix.^24^ This not only weakened the crystallinity of the modified polymer, but also an improvement in the thermodynamically stable β phase, which helped maintain the stability of the melting point of the membrane (detailed in the DSC test in Table 2).

**Table 2:** The phase structure and thermal properties of modified PVDF-CTFE membranes

Membrane code	*F*(β) (%)	*χ*_c_ (%)	*T*_m_ (°C)
M1	79.7	46.5	158.5
M2	86.7	46.2	158.5
M3	87.3	45.6	158.4
M4	88.0	40.9	158.5
M5	87.9	43.3	158.5

**Fig. 6:**
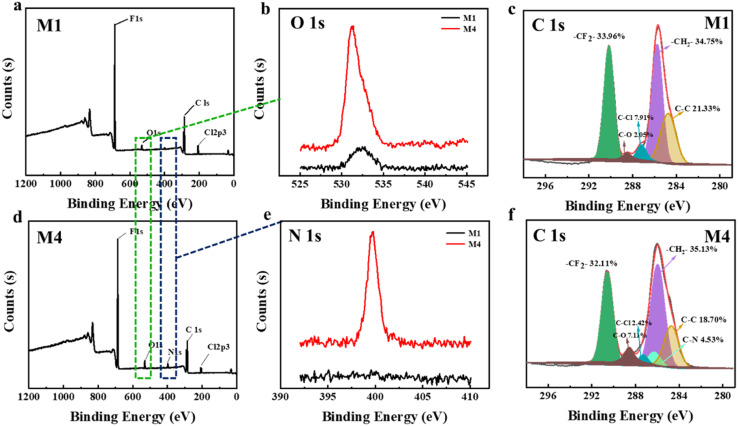
Influence of the addition of *N*-MG on the XPS spectra of the modified porous substrate membranes: (a and c) M1 membrane (blank group), (b) O 1s spectra of M1 and M4 membranes, (d and f) M4 membrane (represents *N*-MG modified membranes), and (e) N 1s spectra of M1 and M4 membranes.

Fig. 6 displays the XPS spectra of the PVDF-CTFE porous membrane surfaces modified with different amounts of *N*-MG. According to the full spectra in Fig. 6(a, b, d and e) the surface of the M1 membrane also exhibited a similar diffraction peak for the O element as observed in the modified M4 membrane. This was because during the preparation of the substrate membrane, PVP was used as a pore-forming agent. As a low molecular weight polymer, PVP was thoroughly mixed and entangled with the PVDF-CTFE segments in the casting solution, and during the phase separation process to form the porous membrane, some of it remained trapped within the membrane.^25,26^ The peak fitting of the XPS spectra of the C 1s peak in Fig. 6(c and f) revealed that the area corresponding to the C–Cl peak in the M4 membrane was smaller than that in the M1 membrane. Additionally, the proportion of C–N and C–O peaks increased, indicating the grafting modification of *N*-MG onto the active C–Cl sites of PVDF-CTFE.

**Fig. 7:**
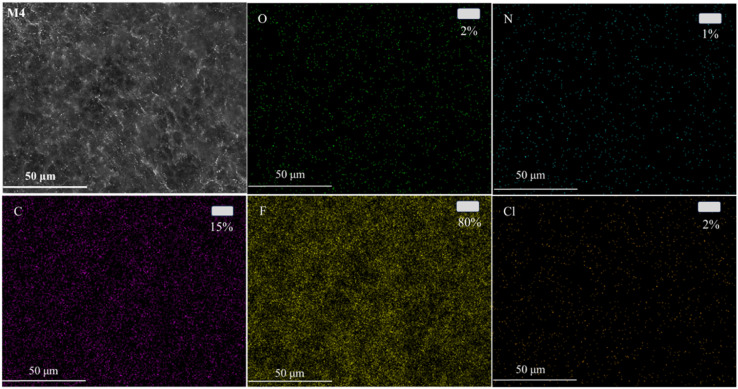
EDS mapping images of the *N*-MG-modified membrane surface (represented by the M4 membrane).

To further analyze the distribution of *N*-MG on the surface of the modified membranes, EDS analysis was conducted using the M4 membrane as an example. The outcomes are displayed in Fig. 7. Besides the C, F, and Cl elements, the PVDF-CTFE porous membrane surface also exhibited a uniform distribution of N and O elements. Combining the above-mentioned analyses, it can be concluded that *N*-MG was not only successfully introduced in the PVDF-CTFE molecular chains through grafting reactions but also uniformly distributed on the substrate membrane surface.

### 3.3 Comprehensive performance of modified substrate membrane

**Fig. 8:**
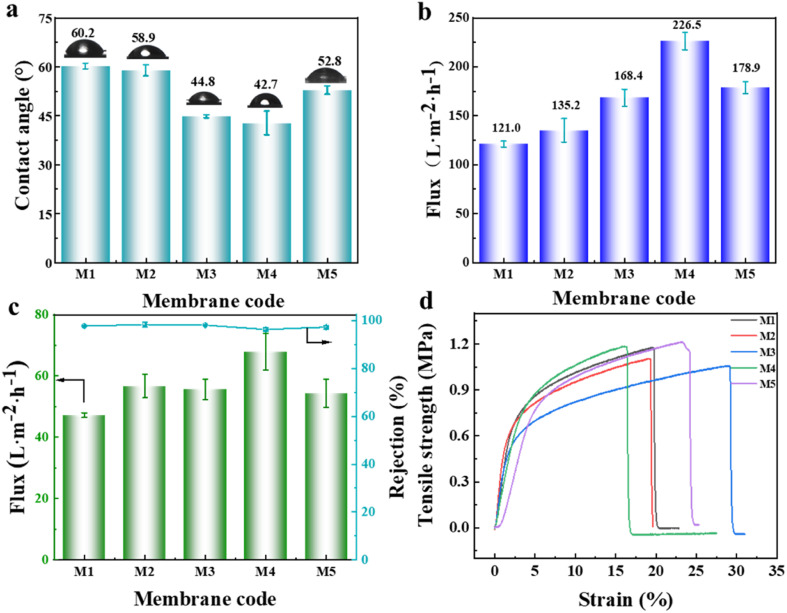
Influence of the addition of *N*-MG on the comprehensive performance of the modified porous substrate membranes: (a) water contact angle, (b) pure water flux, (c) BSA solution flux and rejection rate, and (d) stress–strain curves.

According to the above-mentioned analysis, although the graft modification of the PVDF-CTFE porous membrane with *N*-MG was achieved, to determine whether it can be used as a substrate membrane for NF, comprehensive testing of its properties such as wetting, separation, and stretching needed to be performed. The hydrophilicity of the porous substrate membrane plays a crucial role in the construction and performance of the polyamide separation layer in NF membranes. A smaller water contact angle indicates better hydrophilicity, indicating that the membrane is more easily wetted by aqueous phase solution, which is beneficial for IP.^27,28^ Referring to Fig. 8(a), the surface water contact angle data of the PVDF-CTFE porous membranes modified with different amounts of *N*-MG showed that the addition of *N*-MG, which contained abundant hydroxyl groups, led to an overall decrease in the contact angle. Moreover, when the content of *N*-MG reached 1.5 wt%, the contact angle was the minimum, indicating an improvement in membrane hydrophilicity. Interestingly, when the content of *N*-MG reached 2 wt%, the contact angle did not continue to decrease but slightly increased. Combined with the analysis of the membrane surface morphology (Fig. 3(a)), it can be inferred that the white particles on the membrane surface were not solely undissolved *N*-MG but likely a result of the entanglement between the intrinsic hydrophobic PVDF-CTFE segments and *N*-MG molecular chains.

According to the data presented in Fig. 8(b and c), it was evident that the variations in the flux of pure water and BSA solution were consistent with the trend of membrane porosity (as illustrated in Fig. 4(a)). When the content of *N*-MG reached 1.5 wt%, the flux of the M4 membrane reached the maximum, indicating the best permeability of the membrane at this point. Although the BSA rejection rate of the M4 membrane decreased due to the increase in average pore size, it still maintained a high level of 96%. Fig. 8(d) shows the stress–strain curves of the porous membranes modified with varying amounts of *N*-MG. The tensile breaking strength and elongation fluctuated within a narrow range of 1.0–1.2 MPa and 17–28%, respectively, demonstrating that the mechanical characteristics of the porous membranes were not significantly affected by the introduction of *N*-MG.

### 3.4 Construction of separation layer on modified substrate membrane

**Fig. 9:**
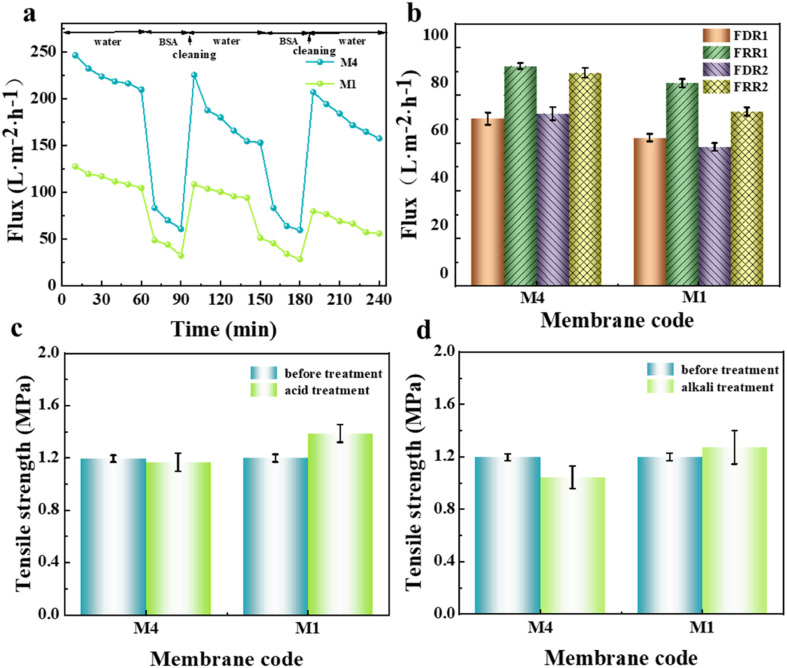
Influence of the addition *N*-MG on the fouling resistance and acid-alkali resistance of the modified porous substrate membranes: (a) dual-cycle test curve for BSA and water, (b) calculated values of FDR and FRR, and (c and d) breaking strength before and after soaking in acid and alkali solution, respectively.

The anti-fouling and acid-alkali resistance of the porous substrate membrane have a very important influence on the formation of the interfacially polymerized separation layer in NF membranes. Good anti-fouling ability can prevent the intrusion of pollutants, and excellent acid–alkali resistance is beneficial for universal IP reaction conditions. Herein, before the IP reaction, the anti-fouling and acid–alkali resistance of the prepared substrate membrane were tested, and the results are shown in Fig. 9. Considering that the primary properties required in porous membrane materials used as NF substrate membranes are hydrophilicity and permeability, the M4 membrane, which exhibited the best comprehensive performance, was selected as the representative. As seen from the dual-cycle testing and calculation results in Fig. 9(a and b), although the modified M4 membrane showed a slight increase in FDR, its FRR was higher than that of the M1 membrane without the addition of *N*-MG. The values of both cycles showed little variation, indicating an improvement in the anti-fouling ability of the membrane after *N*-MG modification. According to the breaking strength data of the substrate membranes obtained before and after immersion in acid and alkali solutions, as shown in Fig. 9(c and d), respectively, it can be observed that the modified M4 membrane, although experiencing a slight decrease, remained within the range of 0.2 MPa, demonstrating its good acid-alkali corrosion resistance.

**Fig. 10:**
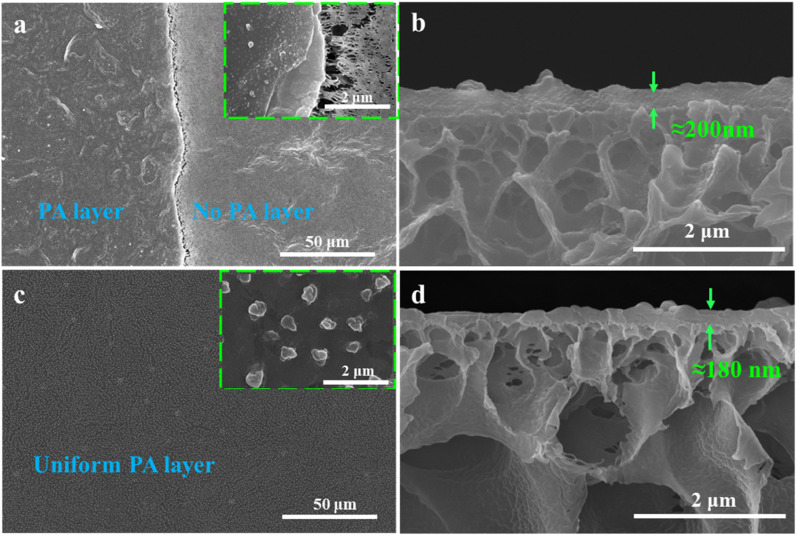
Effect of the addition of *N*-MG to the substrate membranes on the construction of an NF membrane separation layer: (a and b) surface and section morphology of the M1 membrane and (c and d) surface and section morphology of the M4 membrane, respectively.

Based on this, an NF separation layer was constructed through the surfactant-induced IP reaction, using the PVDF-CTFE modified porous membrane surface as the interface. The influence of the addition of *N*-MG to the substrate membranes on the construction of the separation layer was investigated, and the morphology of the prepared composite NF membrane is depicted in Fig. 10. When IP was carried out on the surface of the M1 substrate membrane without the addition of *N*-MG, the formed PA functional layer showed poor uniformity and even separation layer delamination occurred, as shown in Fig. 10(a). Combined with the cross-sectional morphology in Fig. 10(b), the separation layer formed by IP was thick. In contrast, the PA functional layer formed at the surface of the *N*-MG modified M4 membrane was uniform (Fig. 10(c)) and well-bound to the substrate membrane (Fig. 10(d)). Moreover, the addition of the surfactant SDS facilitated the self-assembly of the amphiphilic molecules, promoting the diffusion of amines in the aqueous phase, and thus forming a wrinkled Turing structure.^29–31^ The addition of Na_2_CO_3_ could restrict and regulate the diffusion behavior of amines towards the water–oil interface, enabling the uniform diffusion and polymerization of amines,^32,33^ thereby forming a structurally uniform, smooth and thin PA layer.

**Fig. 11:**
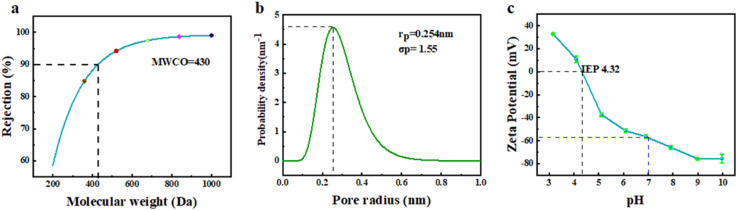
MWCO (a), pore size distribution (b) and surface electronegativity (c) of the NF membrane separation layer constructed by adding Na_2_CO_3_.

To further analyze the structure and properties of the separation layer, the MWCO, pore size distribution and surface electronegativity of the layer prepared by adding Na_2_CO_3_ were tested. The MWCO and average pore size are two important parameters for evaluating the separation performance of NF membranes.^34,35^Fig. 11(a) shows the fitting curve obtained from the rejection test for various molecular weight PEGs using the membrane, and the calculated MWCO was 430, which is in the MWCO range of NF membranes. Fig. 11(b) presents the probability density function of the pore size distribution in the membrane separation layer, with an average pore radius of 0.254 nm. This value is in the range between the radii of monovalent and divalent hydrated ions. Moreover, the size of the majority of pores was mainly distributed in the narrow range of 0.1–0.5 nm, indicating the potential for efficient separation of divalent and monovalent salts.^36^ The surface electronegativity of the separation layer can affect the transport behavior of molecules and ions during the NF process through mechanisms such as charge screening and permeation regulation, thereby achieving the selective separation of solutes. The curve in Fig. 11(c) shows the variation in the surface electronegativity with pH. The membrane surface exhibited strong electronegativity under neutral conditions. During NF separation, substances with positive charges could be adsorbed on the membrane surface through charge screening effects, enabling separation. Furthermore, according to the Donnan equilibrium theory, solvent and ion transport can be influenced by the permeation regulation effect. The isoelectric point of the membrane was found to be at pH = 4.32. Near the isoelectric point, a large flux can usually be achieved, while reducing the adsorption of charged substances, which provides ideas for membrane cleaning.

### 3.5 Separation performance of NF membrane

**Fig. 12:**
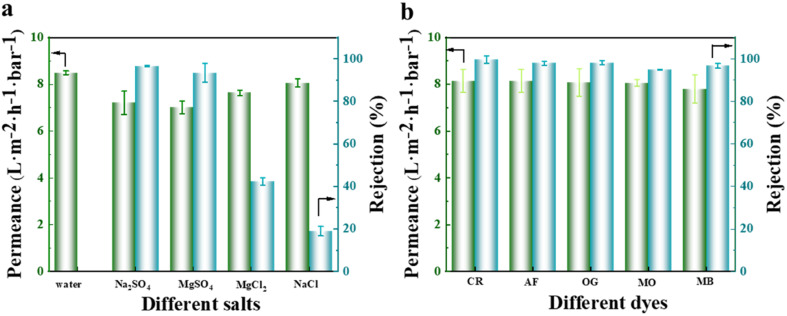
Separation effect of the fabricated NF membrane on various types of salts (a) and dyes (b).

To evaluate the separation efficiency of the NF membrane towards salts and dyes, different types of monovalent and divalent salts, as well as anionic and cationic dyes, were selected as feed solutions during the experiment. The results obtained, as illustrated in Fig. 12(a), revealed that the membrane had a significantly higher rejection rate for Na_2_SO_4_ and MgSO_4_ compared to MgCl_2_ and NaCl. This observation can be explained by the pore size sieving effect, where the hydrated radius of the SO_4_^2−^ ions (divalent ions) was larger compared to that of Cl^−^ ions (monovalent ions).^37^ The NF membrane used in this experiment had a pore size located between the hydrated radii of monovalent and divalent ions (as illustrated in Fig. 11(b)), resulting in a separation efficiency of over 90% for sulfate salts and less than 50% for chloride salts. Notably, the separation efficiency was the lowest for NaCl, which solely consists of monovalent hydrated ions. Additionally, the membrane exhibited a weaker repulsive effect towards MgSO_4_ than towards Na_2_SO_4_, leading to the superior separation performance for Na_2_SO_4_ as a result of the Donnan exclusion effect. The permeate flux of monovalent salts surpassed that of divalent salts due to the permeation-rejection trade-off effect. Concerning the separation data of various dyes, as depicted in Fig. 12(b), the membrane demonstrated an excellent separation performance for all dyes, achieving a rejection rate of over 98% for anionic dyes (CR, AF, and OG).

**Fig. 13:**
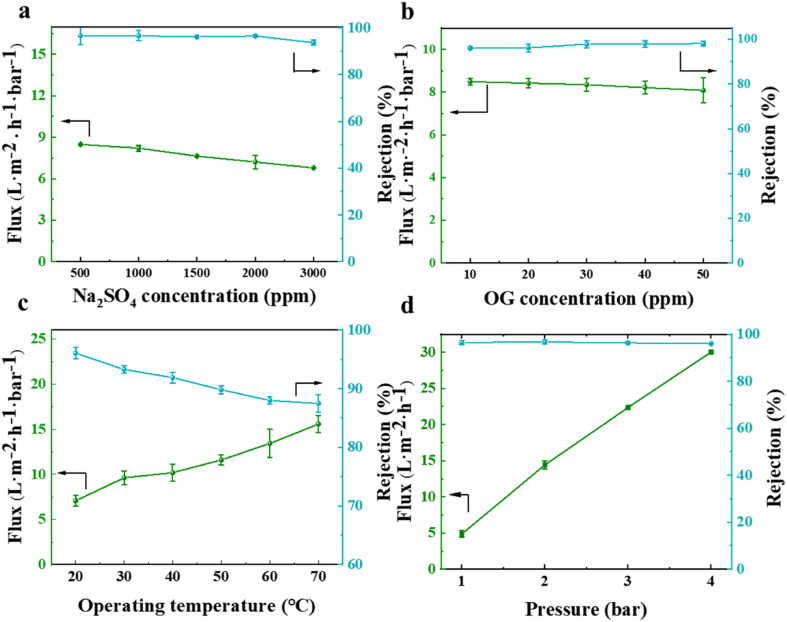
Effect of different salt (a) and dye (b) solution concentrations and different test temperatures (c) and pressures (d) on the separation performance of the prepared NF membrane.

Besides testing the filtration and separation of different salt and dye solutions by the NF membrane, the effects of different salt (represented by Na_2_SO_4_) and dye (represented by OG) concentrations, as well as different test temperatures and pressures (using a feed solution containing 2000 ppm Na_2_SO_4_) were also studied. The results, as depicted in Fig. 13(a and b), indicate that an increase in the concentration of salt or dye solution led to a decrease in the permeate flux. This was because more salt ions or dyes accumulated on the membrane surface, increasing the electro-viscous effect and pore blockage. The rejection rate did not significantly change with an increase in the feed solution concentration, indicating its excellent and superior separation performance. In terms of the impact of feed solution temperature and test pressure on the membrane separation performance, as illustrated in Fig. 13(c and d), it was observed that the permeate flux generally increased in proportion to the elevation in temperature and pressure, respectively. A higher temperature intensified the movement of salt ions in the solution, leading to a lower salt rejection rate. However, the rejection rate still remained above 85%, indicating that the prepared NF membrane exhibited strong high-temperature resistance. When the test was conducted at pressures below 4 bar, the salt rejection rate remained relatively constant.

### 3.6 Stability and selective separation performance of NF membrane

**Fig. 14:**
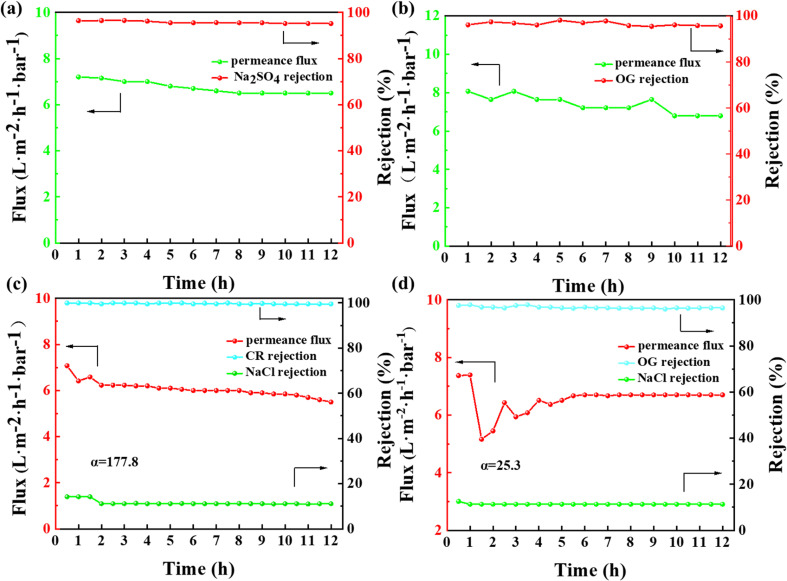
. Separation efficiency of salt solution (a), dye solution (b) and mixed solution of salt and two different dyes (c: CR and d: OG) of prepared NF membrane continuously for 12 h.

To investigate the operational stability and selective separation performance of the membrane towards dyes/salts, continuous NF tests were conducted on the membrane for a duration of 12 h using salt solution (2000 ppm Na_2_SO_4_), dye solution (50 ppm OG), and a mixture of salt and dye solution.^38^ The results, as depicted in Fig. 14(a and b), indicate that the rejection rates of salts and dyes remained essentially unchanged with an increase in operating time, demonstrating the excellent separation stability, respectively. The permeate flux of salts and dyes exhibited a slight decline. This can be attributed to the increasing concentration of salts or dyes on the membrane feed side as the separation process progressed. This led to an increase in the solute concentration gradient at the membrane surface, which exceeded the concentration in the solution, thereby affecting the flux because of the concentration polarization phenomena.^39^ The Na_2_SO_4_ rejection rates of the prepared NF membranes were compared with some recently reported PVDF NF membranes as shown in Table 3.

**Table 3:** Comparison of Na_2_SO_4_ separation effect with reported studies

Membrane	Na_2_SO_4_ content (ppm)	Pressure (MPa)	Permeability (L per m^2^ per h per bar)	Rejection rate (%)	Reference
10% S-PVDF	2000	0.35	1.9	97.0	40
PA nfm	1000	—	13.1	98.3	41
MT-0.75%	1000	0.4	21.5	98.6	42
CMC-Na/PVDF	1000	0.4	2.7	88.5	43
PVDF/CMC-ZnO(M0.05)	1000	1.0	13.5	95.0	44
TFC PA	5000	3.0	0.5	96.1	45
PVDF-CTFE@*N*-MG	2000	0.2	7.2	96.5	This work

Finally, a mixed solution simulating dyeing and printing wastewater was prepared using 50 ppm CR and OG dyes, and 2000 ppm NaCl salt, respectively. The fabricated NF membrane was tested for its selective separation performance towards dyes/salts (as depicted in Fig. 14(c and d)), respectively. In addition to verifying the long-term operational stability of the membrane, the selective separation of dyes and salts was achieved. The calculated selectivity coefficients for CR/NaCl and OG/NaCl were 177.8 and 25.3, respectively. The difference in the selective separation performance for different dyes is mainly attributed to their chemical properties. CR is an alkaline dye, and its separation performance is enhanced by pore sieving and the Donnan effect. Alternatively, OG is a smaller organic dye with strong solubility, which readily forms homogeneous solutions with water. During the separation process, it is difficult for an adequate retention layer to be formed on the membrane surface, thereby affecting the separation efficiency. A comparison with recent reported studies showed that the fabricated NF membrane exhibited certain selectivity performance but further improvement in separation efficiency was still needed through structural design (Table 4).

**Table 4:** The selective separation effects of dye/NaCl compared with reported studies

Membranes	Type of dyes	Operating pressure (MPa)	Permeability (L per m^2^ per h per bar)	Dye rejection rate (%)	NaCl rejection rate (%)	*α*	Reference
PVDF/CD-0.75/25	Congo red	0.4	9.2	99.0	6.8	93.2	46
Modified PVDF/SMANa membrane	Congo red	0.2	251.6	99.9	1.7	983	47
PVDF 5-5	Congo red	0.4	26.4	99.4	14.9	851	48
ZIF-8/PVDF	Congo red	0.1	24.5	99.0	2.0	98	49
PA/PVDF	Congo red	0.2	10.2	100	6.2	9380	50
PVDF-CTFE@*N*-MG	Orange G	0.2	6.7	96.5	11.3	25.3	This work
PVDF-CTFE@*N*-MG	Congo red	0.2	6.2	99.5	11.1	177.8	This work

## 4. Conclusions

In this study, a PVDF-CTFE porous substrate membrane was modified by introducing *N*-MG to enhance its hydrophilicity and permeability, leading to the successful preparation of a composite NF membrane with outstanding performance. The FTIR, XPS, and EDS analyses revealed the effective grafting of *N*-MG on the surface of the substrate membrane. The morphological characterization indicated that the introduction of *N*-MG increased the number of micropores on the surface of the porous substrate membrane and formed a uniformly structured PA functional layer. Regarding the separation performance, the fabricated NF membrane displayed excellent rejection rates for divalent salts, particularly the optimal separation efficiency for sulfate salts. Simultaneously, during the separation process of dyes, the membrane demonstrated excellent selectivity, achieving a rejection rate of over 98% for anionic dyes. Furthermore, the NF membrane demonstrated good long-term operational stability, given that its rejection rates for salts and dyes remained relatively constant during continuous separation experiments lasting 12 h. The test results of the mixed solution simulating dyeing and printing wastewater showed that the NF membrane achieved the selective separation of dyes and salts with high selectivity coefficients. In summary, this study successfully fabricated a composite NF membrane based on modified PVDF-CTFE, which exhibited a favorable morphology, chemical composition, and comprehensive performance. This presents novel ideas and methodologies for the preparation and application of NF membranes. Future research can further optimize the structure and performance of membranes to meet broader application demands.

## Author contributions

Xinyu Pan: investigation, data curation, visualization, writing – original draft, writing – review & editing. Jian Pan: conceptualization, methodology, project administration, funding acquisition, writing – original draft, writing – review & editing. Zhuoqun Li: data curation, validation, writing – review & editing. Wenqiang Gai: data curation, investigation. Guangshun Dong: formal analysis, software. Min Huang: visualization, formal analysis. Lilan Huang: methodology, resources, supervision, writing – review & editing.

## Conflicts of interest

The authors affirm that there are no known financial conflicts of interest or personal relationships that could have potentially influenced the outcome of this research.

## Supplementary Material

RA-014-D4RA00359D-s001
