# Supplementary material for: Preparation of N-MG-modified PVDF-CTFE substrate composite nanofiltration membrane and its selective separation of salt and dye
Source: RSC Adv. 2024 Apr 18;14(17):11992–2008. doi: 10.1039/d4ra00359d (PMC11024597; doi:10.1039/d4ra00359d)
Supplement: RA-014-D4RA00359D-s001 [file RA-014-D4RA00359D-s001.pdf]

Electronic Supplementary Material (ESI) for RSC Advances.
This journal is © The Royal Society of Chemistry 2024

Supplementary Information

Preparation of N-MG modified PVDF-CTFE substrate composite nanofiltration membrane and its selective separation of salt and dye

Xinyu Pan [a], Jian Pan [a, *], Zhuoqun Li [a], Wenqiang Gai [a], Guangshun Dong [a], Min Huang [a], Lilan Huang [a, *]

a- School of Materials Science and Engineering, Shandong University of Technology, Zibo 255000, China

*Corresponding author. E-mail address: panjian@sdut.edu.cn (J. Pan);

huanglilan_sdut@163.com (L. Huang)

2.4 Membrane characterization

2.4.1 Membrane morphology

The surface and cross-section morphology of the membranes were evaluated by field emission SEM (FE-SEM) (Quanta 250, FEI Co., USA) under standard high vacuum conditions at 3.00 kV. The elemental composition and distribution of C, F, Cl, O and N on the surface of the membrane were analyzed using EDS within a 150×120 μm area. The membranes were fractured in liquid nitrogen, which facilitated obtaining complete and clear cross sections. Due to the poor electrical conductivity of the polymer, the samples were affixed to the sample stage using a conductive adhesive and subsequently coated with gold under vacuum conditions.

2.4.2 Porosity and pore size distribution

Porosity was determined by the gravimetric method. Firstly, a circular membrane with a diameter 1.5 cm was carefully cut out and any water present on its surface was blotted dry with filter paper. The weight of the wet membrane (M_w) was then measured using an analytical balance. The membrane was subsequently dried in a vacuum drying oven maintained at 50° C until it achieved a constant weight, after which the weight of the dry membrane (M_d) was measured. Finally, the porosity (ε) of the membrane was calculated using the following formula [1]:

$$\varepsilon(\%) = \frac{(M_w - M_d)/\rho_w}{(M_w - M_d)/\rho_w + M_w/\rho_d} \times 100 \tag{1}$$

where ρ_w and ρ_d represent the density of water (1.0 g·cm^{-3}) and polymer (1.78 g·cm^{-3}), respectively.

The pore size distribution and average pore size of prepared membranes were determined using the bubble-pressure method by a pore diameter analyzer (BSD-PB, Beishide Instrument Technology (Beijing) Co. Ltd., China).

2.4.3 Physicochemical structure

The effect of N-MG addition on the physicochemical structure of the membranes was assessed by comparing their infrared spectra. The spectra were recorded in attenuated total reflection mode within the range of 600 to 4000 cm^{-1} using FT-IR (Nicolet 5700, Thermo Fisher Co., USA) with a resolution of 4 cm^{-1}. Typically, the β-phase content of the polymer matrix ($F(\beta)$) was calculated by the following equation [1,2]:

$$F(\beta)(\%) = \frac{A_\beta}{\left(K_\beta/K_\alpha\right)A_\alpha + A_\beta} \times 100 \tag{2}$$

where A_α and A_β represent the absorbance of the α-phase (760 cm^{-1}) and β-phase (840 cm^{-1}) respectively, and their absorption coefficients are K_α = 6.1 × 10^4 cm^2·mol^{-1} and K_β = 7.7× 10^4 cm^2·mol^{-1}.

XPS (K-Alpha, Thermo Fisher Co., USA) analysis was performed to characterize the surface chemical elements of the PVDF-CTFE membrane. The XPS measurement involved a broad-spectrum scan across electron binding energies ranging from 0 to 1350 eV with a resolution of 1 eV. Additionally, a narrow spectrum scan was conducted specifically on the C1s region for qualitative and semi-quantitative analysis.

The thermal properties of each membrane were examined using DSC (Q100, TA Co., USA). Approximately 5 mg of each membrane was placed into a sample tray and heated from room temperature to 250 ° C at a heating rate of 10 ° C·min^{-1} under a nitrogen atmosphere. The heat flow data during the heating process were recorded. To account for the influence of N-MG on crystallinity, the following equation was employed to calculate the membrane crystallinity (χ_c) [1,2]:

$$\chi_c(\%) = \frac{\Delta H_m}{x(\Delta H_{100\%})_\alpha + y(\Delta H_{100\%})_\beta} \times 100 \tag{3}$$

where x and y are the mass fraction of α-phase and β-phase crystal, respectively. ΔH_m denotes the

recorded melting enthalpy of the polymer, while the melting enthalpy of the α-phase and β-phase is represented by $(\Delta H_{100\%})_{\alpha}$ = 93.04 J·g^{-1} and $(\Delta H_{100\%})_{\beta}$ = 103.4 J·g^{-1}, respectively.

2.5 Membrane performance test

2.5.1 Contact angle of porous substrate membrane

To measure the static contact angle of the membrane samples, a contact angle measuring instrument (JY-82A, Ding Sheng Co., China) was utilized. The membrane samples were appropriately sized and fixed onto slides. The droplet volume was set to 1 μL, and contact measurements were carried out to capture images and record data. The experimental data was generated by performing measurements from at least five different positions and calculating the average value.

2.5.2 Mechanical properties of porous substrate membrane

Prior to testing, the membrane sample was prepared by cutting it into long strips measuring 10 cm in length and 2 cm in width. The thickness of the sample was then measured using a vernier caliper (taking the average of 5 measurements at different points) to determine the effective cross-sectional area.

The prepared sample was affixed to the testing machine (YG001 N+, Hong Da Co., China), and a tensile speed of 5 mm·min^{-1} was applied to determine the elongation at break and breaking strength. This process was repeated 5 times for each sample to obtain an average value.

2.5.3 Zeta potential of separating layer

The surface zeta potential of the membrane was tested by a solid-state zeta potential analyser (Exceed 3, Anton Paar, Austria). For the measurements, a 1 mM KCl electrolyte solution was selected, and pH adjustments were made using 0.05 M HCl (acidic) or 0.05 M NaOH (basic) solutions within the range of 3.0 to 10.0.

2.5.4 MWCO and pore size distribution of functional layer

The MWCO of the NF membrane was measured by using 1000 ppm PEG solutions with different molecular weights (200, 400, 600, 800, and 1000 Da). The selected PEG solutions were passed through the membrane. The PEG concentrations of the permeate solution and the feed solution were analyzed by the total organic carbon analyzer (TOC-L, Shimadzu, Japan). When the rejection rate for a particular PEG solution reached 90%, the corresponding molecular weight was considered as the MWCO of the NF membrane [3,4].

Assuming no steric and hydrodynamic interaction between the PEG solute and the membrane pores, the pore diameter distribution of the NF membrane could be obtained through the following probability density function:

$$\frac{dR(r_p)}{dr_p}=\frac{1}{r_p \ln \sigma_p \sqrt{2\pi}}exp\left[-\frac{\left(\ln r_p - \ln \mu_p\right)^2}{2\left(\ln \sigma_p\right)^2}\right] \tag{4}$$

where μ_p is the mean pore diameter which was determined at the PEG rejection of 50% and σ_P is the geometric standard deviation, which is defined as the ratio of r_p at the PEG rejection of 84.13% over that of 50%. Where r_p represents Stokes radius of PEG was calculated by the following equations:

$$r_p = 16.73 \times 10^{-12} \times M^{0.557} \tag{5}$$

where M is molecular weight of PEG solute.

2.5.5 Separation performance test

The tests were carried out using lab-made equipment with an effective membrane area of 7.065 cm^2. In the pure water flux and ultrafiltration performance testing of the porous substrate membrane, deionized water was initially used as the test solution. The membrane was pre-pressurized at 0.15 MPa for 30 min, and once the water permeate reached a stable flow rate, the experiment was conducted under a pressure of 0.1 MPa. The volume of permeate and the corresponding time were recorded. The calculation formula for pure water flux (J) is as follows:

$$J = V/S \times t \tag{6}$$

where V is the permeate volume (L), S is the effective membrane area (m^2), and t is the operation time (h).

Then, a 1 $g \cdot L^{-1}$ solution of BSA was used as the test solution. The filtration test was conducted under a pressure of 0.1 MPa, and the permeate volume was recorded every 10 min. The flux was calculated using formula (6). The absorbance of BSA in the feed and permeate solutions at 280 nm were measured using an ultraviolet-visible (UV) spectrophotometer (UV-1100, Mapada, China), and the BSA concentration was calculated. The rejection rate (R) of BSA was calculated using the following formula:

$$R(\%) = \left(1 - \frac{C_p}{C_f}\right) \times 100 \tag{7}$$

where C_p and C_f represent the solute concentration in the permeate and feed solutions, respectively.

The anti-fouling ability of the porous substrate membranes was evaluated through a dual-cycle filtration testing. The specific procedure was as follows: the pre-pressed membrane underwent a 60-minute pure water flux test, followed by a 30-minute BSA rejection test. After ultrasonic cleaning, the membrane underwent a second cycle of pure water flux and BSA rejection testing. Finally, after ultrasonic cleaning, the recovery of pure water flux was measured. Flow decline ratio (*FDR*) and flow recovery ratio (*FRR*) parameters are calculated by the following formula respectively [5].

$$FDR(\%) = (1 - \frac{J_b}{J_{W1}}) \times 100 \tag{8}$$

$$FRR(\%) = (\frac{J_{W2}}{J_{W1}}) \times 100 \tag{9}$$

where J_b is the BSA flux, J_{w2} is the pure water flux after cleaning with water and J_{w1} is the pure water flux before fouling.

The acid and alkali resistance of the membranes was tested by immersing the prepared porous substrate membranes in a HCl solution (pH=3) and NaOH solution (pH=11), respectively, for 10 days, followed by rinsing [6-8]. The fracture strength of the membranes before and after immersion was measured using an electronic single fiber strength machine. All membranes were tested three times and the average was taken as the final result.

In the testing of separation performance of prepared TFC NF membranes, the membrane was first pre-pressed with deionized water at 0.4 MPa for 30 min. Then, filtration separation tests were conducted at a pressure of 0.2 MPa using solutions of salts (2000 ppm Na_2SO_4, $MgSO_4$, $MgCl_2$ and NaCl) and dyes (50 ppm CR, AF, OG, MO, MB). The filtrate was collected every 10 min, and the volume and solute concentration were tested. The permeate flux of the NF membranes for salt and dye solutions was calculated using Formula (6). The concentration of salts and dyes in the filtrate and feed solution was measured using a conductivity meter (DDS-307, Shanghai Yueping, China) and an UV spectrophotometer, respectively, with the maximum absorption wavelengths for different dyes being 498 nm (CR), 540 nm (AF), and 475 nm (OG), 464 nm (MO), 664 nm (MB). Finally, the rejection rates of the NF membranes for different salts and dyes were obtained using Formula (7).

2.5.6 Stability and selective separation performance

The stability performance of the NF membrane was evaluated using the same NF test setup. The membrane was pre-pressurized at 0.4 MPa for 30 min, followed by a 12 h testing period at 0.2 MPa. Every half hour, the filtrate was taken for testing, and the permeation flux and rejection were calculated.

In this experiment, a mixture of dye and salt was used to simulate printing and dyeing wastewater. The NF separation test was operated by using CR/NaCl and OG/NaCl mixture solutions for 12h. The concentrations of salt and dye were 2000 ppm and 50 ppm, respectively. The selectivity (α) of dyes to NaCl was calculated by the following formula [9]:

$$\alpha = \frac{1 - R_A}{1 - R_B} \tag{10}$$

where R_A and R_B represent the rejection rate of NaCl and dye, respectively.

Fig. S1. Digital photos of the effect of N-MG addition on membrane casting solution.

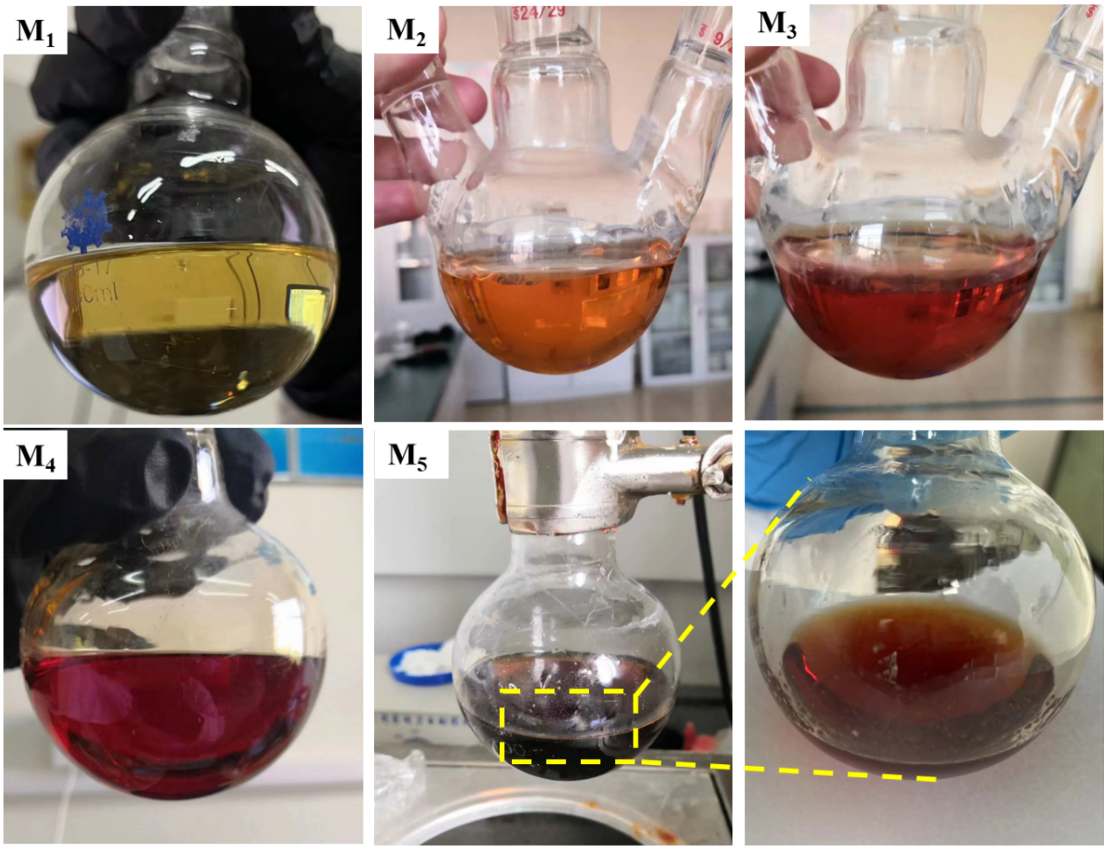

Fig. S1. Digital photos of the effect of N-MG addition on membrane casting solution.

As can be seen from Fig. S1, with the increase of N-methylglucamine (N-MG) addition, the color of the casting solution gradually deepened. When the N-MG addition reached 2 wt.%, the casting solution appeared dark brown and there was some undissolved material liquid on the wall of the flask. Analysis with an electron microscope indicated that at this point, the N-MG addition had reached its maximum and the dissolution was insufficient.

References

[1] L. Zheng, J. Wang, D. Yu, Y. Zhang, Y. Wei, Preparation of PVDF-CTFE hydrophobic membrane by non-solvent induced phase nversion: relation between polymorphism and phase inversion, J. Membr. Sci. 550 (2018) 480–491. https:// doi.org/10.1016/j.memsci.2018.01.013.

[2] L. Zheng, J. Wang, Z. Wu, J. Li, Y. Zhang, M. Yang, Y. Wei, Preparation of interconnected biomimetic Poly(vinylidene fluoride-co-chlorotrifluoroethylene) hydrophobic membrane by tuning the two-stage phase inversion process, ACS Appl. Mater. Inter. 8 (2016) 32604–32615. https://doi.org/10.1021/ acsami.6b11249.

[3] B. Sutariya, S. Karan, A realistic approach for determining the pore size distribution of nanofiltration membranes, Separ. Purif. Technol. 293 (2022) 121096. https:// doi.org/10.1016/j.seppur.2022.121096.

[4] S. Karan, Z. Jiang, A.G. Livingston, Sub-10 nm polyamide nanofilms with ultrafast solvent transport for molecular separation, Science. 348 (2015) 1347–1351. https:// doi.org/10.1126/science.aaa5058.

[5] X. Kong, Y. Sun, X. Lu, C. Wu, Facile preparation of persistently hydrophilic poly(vinylidene fluoride-co-trifluorochloroethylene) membrane based on in-situ substitution reaction, J. Membr. Sci. 609 (2020), 118223, https://doi.org/10.1016/j.memsci.2020.118223.

[6] C. Zhang, Y. Min, Y. Bai, J. Gu, Y. Sun, Fabrication and characterization of chlorinated polyvinyl chloride microporous membranes via thermally induced phase separation process, J. Appl. Polym. Sci.134 (2016), 44346, https://doi.org/10.1002/APP.44346.

[7] W. Ma, W. Ren, X. Bai, J. Pan, L. Huang, Q. Huang, Z. Guo, X. Wang, Facile fabrication of PVDF-CTFE microporous membranes with optimized surface and sublayer structure via non-solvent induced phase separation, J. Water Process Eng. 45 (2022) 102504. https:// doi.org/10.1016/j.jwpe.2021.102504.

[8] W. Ren, J. Pan, W. Gai, X. Pan, H. Chen, J. Li, L. Huang, Fabrication and characterization of PVDF-CTFE/SiO_2 electrospun nanofibrous membranes with micro and nano-rough structures for efficient oil-water separation, Sep. Purif. Technol. .311 (2023) 123228. https:// doi.org/ 10.1016/j.seppur.2023.123228.

[9] Y. Jiang, S. Li, J. Su, X. Lv, S. Liu, B. Su, Two dimensional COFs as ultra-thin interlayer to build TFN hollow fiber nanofiltration membrane for desalination and heavy metal wastewater treatment, J. Membr. Sci. 635 (2021). https:// doi.org/ 10.1016/j.memsci.2021.119523.
